# Depressive symptoms and cognitive impairment: A 10-year follow-up study from the Survey of Health, Ageing and Retirement in Europe

**DOI:** 10.1192/j.eurpsy.2021.2230

**Published:** 2021-08-27

**Authors:** Fei-Fei Han, Hui-Xin Wang, Jia-Jia Wu, Wu Yao, Chang-Fu Hao, Jin-Jing Pei

**Affiliations:** 1 College of Public Health, Zhengzhou University, Zhengzhou, Henan, China; 2 Stress Research Institute, Department of Psychology, Stockholm University, Stockholm, Sweden

**Keywords:** Depressive symptoms, dose–response, late-life, mild cognitive impairment

## Abstract

**Background:**

Depressive symptoms and cognitive impairment often coexisted in the elderly. This study investigates the effect of late-life depressive symptoms on risk of mild cognitive impairment (MCI).

**Methods:**

A total of 14,231 dementia- and MCI free participants aged 60+ from the Survey of Health, Ageing, and Retirement in Europe were followed-up for 10 years to detect incident MCI. MCI was defined as 1.5 standard deviation (SD) below the mean of the standardized global cognition score. Depressive symptoms were assessed by a 12-item Europe-depression scale (EURO-D). Severity of depressive symptoms was grouped as: no/minimal (score 0–3), moderate (score 4–5), and severe (score 6–12). Significant depressive symptoms (SDSs) were defined as EURO-D score ≥ 4.

**Results:**

During an average of 8.2 (SD = 2.4)-year follow-up, 1,352 (9.50%) incident MCI cases were identified. SDSs were related to higher MCI risk (hazard ratio [HR] = 1.26, 95% confidence intervals [CI]: 1.10–1.44) in total population, individuals aged 70+ (HR = 1.35, 95% CI: 1.14–1.61) and women (HR = 1.28, 95% CI: 1.08–1.51) in Cox proportional hazard model adjusting for confounders. In addition, there was a dose–response association between the severity of depressive symptoms and MCI incidence in total population, people aged ≥70 years and women (*p*-trend <0.001).

**Conclusions:**

Significant depressive symptoms were associated with higher incidence of MCI in a dose–response fashion, especially among people aged 70+ years and women. Treating depressive symptoms targeting older population and women may be effective in preventing MCI.

## Introduction

As life expectancy continues to rise, dementia incidence is exponentially increasing [1]. It has reported that nearly 43.8 million people suffering from dementia worldwide in 2016 [2], this figure is anticipated to be tripled [3] and will cost $4 trillion by 2050 [4], placing a heavy burden on individuals, families, and societies. Because of the long latent period before the clinical manifestation of dementia [5], increasing attention is being paid to the prodromal stage of dementia-mild cognitive impairment (MCI) in recent years. MCI is characterized by subtle but measurable decline in cognitive abilities, whereas daily activities are not significantly affected [6]. Studies have found that people with MCI are at greater risk of dementia, with the annual progression rates 3–5% in community-based populations [7] and 10–15% in clinical settings [8]. Thus, identifying modifiable risk factors are essential because of the lack of effective treatment nowadays [9].

A growing body of evidence support that depression is one of the modifiable risk factors for dementia [9], which is characterized by progressive decline in cognitive function [10,11]. Depressive symptom and cognitive impairment often co-occur in advanced age, leading to a variety of emotional and physical problems that affect the ability of individuals in work and life and further reduce the quality of life [12,13]. However, whether or not depressive symptom is a risk factor for MCI remains unclear. Most studies have reported that depression is common in patients with MCI [14,15], being a predictor of MCI or progressive cognitive decline [16–18], whereas other studies have reported that depression is not associated with MCI [19,20].

The occurrence of depression has been reported to increase with age, peaking at 60–64 years old, but declining among people over 80 years old [21]. However, in the existing studies, the study samples have been limited to either middle-aged [22] or clinical patients [23], which are not representative to general older population. Second, some of the previous studies were cross-sectional [24,25], and the existing longitudinal studies have either limited follow-up time [26] or limited sample size [27], which are insufficient to determine the association between depressive symptoms and MCI. Further, the potential dose–response relationship between depression severity and cognitive impairment from general population has rarely been investigated. Among them, two studies demonstrated that the higher the baseline depression severity, the greater the subsequent global cognitive impairment [28,29], and one study found that this dose–response relationship was retained in processing speed, not in episodic memory or executive function [30], whereas other two studies did not found such a relationship in global cognition [31,32].

Using data from a population-based investigation, the Survey of Health, Ageing and Retirement in Europe (SHARE), the current study aims to address: (a) the influence of significant depressive symptoms (SDSs) on incidence of MCI; (b) whether there exists a dose–response association between the severity of depressive symptoms and MCI; and (c) whether these relationships vary by age and gender.

## Method

### Study population

SHARE is a biennial longitudinal survey of the aging process in individuals aged 50+ from 11 European countries in 2004 and Israel in 2005. So far, it has conducted six panel waves (waves 1, 2, 4–7) and an ongoing wave (wave 8), with a retrospective data in wave 3 (SHARELIFE). The investigation was mainly focused on health, socio-economic status, and social and family networks, the detailed information could be found elsewhere (http://www.share-project.org/). Participants were interviewed using standardized computer-assisted personal interview by trained staff [33].

In the present study, we chose wave 2 [34] as baseline survey and waves 4 [35], 5 [36], 6 [37], and 7 [38] as follow-up surveys because dementia status was not accessed in waves 1 [39] and cognitive tests were not performed in wave 3 [40]. Ethic permission of SHARE was approved by the University of Mannheim’s internal review board (IRB) and Ethics Council of the Max Planck Society. Each subject signed informed consent, including the storage and the use of relevant data [41].

**Figure 1.**
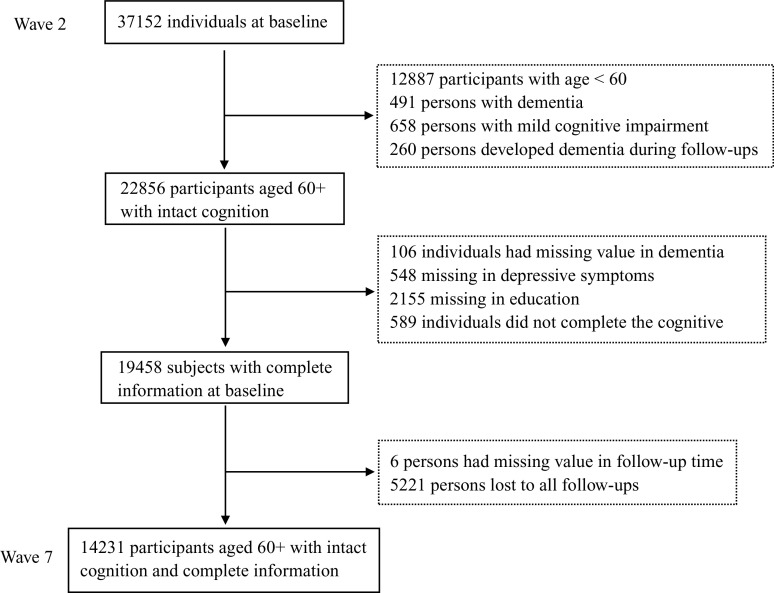
Flowchart of sample selection process.

The SHARE wave 2 investigated 37,152 participants. Of these, we excluded those younger than 60 years (*n* = 12,887), with a diagnosis of dementia (*n* = 491) or MCI (*n* = 658), and those who developed dementia during follow-ups (*n* = 260). We further excluded those with missing information on baseline dementia diagnosis (*n* = 106), depressive symptoms (*n* = 548), educational attainment (*n* = 2155), cognitive function (*n* = 589), follow-up time (*n* = 6), and those who were lost to all follow-ups (*n* = 5,221). After the exclusion, 14,231 individuals were included in the analysis (Figure 1).

### Assessment of depressive symptoms

Depressive symptoms were assessed by a self-reported European-Depression (EURO-D) [42] scale. The structured scale covers 12 emotional states: depressed mood, pessimism, suicidal tendencies, guilt, sleep, interest, irritability, appetite, fatigue, concentration, enjoyment, and tearfulness in the past month. Each item has a yes (score 1) or no (score 0) answers, the sum scores ranged from 0 to 12, with higher scores indicating greater severity of depressive symptoms. The EURO-D scale has good internal consistency [43] (Cronbach’s alpha ranging from 0.78 to 0. 95) and external validity [44]. SDSs were defined as EURO-D score ≥ 4 and used in the analysis as dichotomized variable (SDSs vs. non-SDSs) [45]. This cut-off has been shown to have a reasonable sensitivity (63–83%) and specificity (49–95%) in 14 European countries [46]. To further explore the dose–response association between the severity of depressive symptoms and MCI incidence, the participants were divided into three groups: (a) no/minimal (score 0–3), (b) moderate (score 4–5), and (c) severe depressive symptoms (score 6–12).

### Assessment of cognitive function

Assessments of cognitive function were performed at each wave including: episodic memory, executive function, and language. Episodic memory consists of immediate recall and delayed recall of a 10-word list based on a modified version of the Rey Auditory Verbal Learning Test [47]. Two tests were required to recall in 1 and 5 min later with a full score of 10, respectively. Executive function and language were assessed with verbal fluency test, asking respondents to correctly name as many animals as possible within 1 min. For each domain, higher scores indicate better cognition.

Because of the heterogeneity among individuals, we used age- and education-standardized cognitive scores for the analysis. Step 1, the mean and standard deviation (SD) for immediate recall, delayed recall, and verbal fluency were calculated, respectively, according to the resulting 15 categories of the cross-tabulation of age- (60–64, 65–69, 70–74, 75–79, and over 80) and education- (low, middle and high). Step 2, to facilitate comparison, the *z*-score of each cognitive domain was calculated using the raw score subtracting the mean and dividing by the SD of the corresponding categories [48]. To ensure the comparability across waves, the cognitive scores at follow-ups were also standardized using the same formula and grouping as in step 1. The global cognitive score was calculated as the average *z*-score of all the three cognitive domains. MCI was defined as 1.5 SD below the mean of the standardized global cognition score [49].

### Assessment of covariates

Information on age, gender, country, marital status and living arrangement, educational attainment, smoking, alcohol consumption, physical activity, body mass index (BMI), the number of chronic diseases, history of affective or emotional disorders, and anti-anxiety or depression medication were collected at baseline. Education attainment was ascertained following the International Standard Classification of Education-97 (ISCED-97) and divided into low (ISCED score 0–2), middle (ISCED score 3–4), and high (ISCED score 5–6) [50]. Missing information in baseline education was replaced with values from other waves because their levels of education should be constant. Marital status was obtained by asking the participants: “What is your marital status?” and living arrangement by the question: “Do you have a partner who lives outside this household?,” these two variables were integrated into one and was categorized into living with a partner (married and living with spouse or registered partnership in the same household) and living alone (married but not living with spouse, never married, divorced, or widowed) as in previous studies [51].

Smoking status was dichotomized as non/ex-smokers (never smoked or stopped smoking currently) and current-smokers (currently smoking). Alcohol assumption was classified into frequent-drinkers (five or more units of alcohol a week), usual-drinkers (more than twice a week), and nondrinkers (less than twice a month or not at all) during the last 3 months. Physical activity was dichotomized as inactive (never vigorous nor moderate physical activity) and active (participated in moderate or vigorous exercise at least once a week or 1–3 times a month). BMI was divided into four groups: underweight (<18.5 kg/m^2^), normal (18.5–24.9 kg/m^2^), overweight (25.0–29.9 kg/m^2^), and obese (≥ 30.0 kg/m^2^). The number of chronic diseases was the sum of these diseases: heart attack, hypertension, high blood cholesterol, stroke, diabetes, lung disease, asthma, osteoporosis, arthritis, cancer, peptic ulcer, Parkinson disease, cataracts, hip fracture, and femoral fracture, except dementia because it was related to the outcome (MCI), and it was grouped into four categories (0 = none, 1 = few, 2–3 = some, and 4 or above = many).

### Statistical analyses

The differences in baseline characteristics between participants with different depression status were shown in frequency and percentage for categorical variables and median with interquartile range (IQR) for continuous variables. The baseline characteristics of the nonparticipants due to missing information were compared with the study sample in terms of *N* (%) for categorical variables and mean ± SD for continuous variables.

Multivariate Cox proportional hazard models were conducted to examine the association of baseline SDSs and the severity of depressive symptoms with incidence of MCI. The follow-up time (year) was calculated as the dates between the baseline interviews and the occurrence of MCI at the first time, or the last follow-up examination for participants with normal cognition during the follow-up period. The proportional hazard assumption in Cox regression models across all covariates was assessed, and the covariates that satisfy the assumption were retained in the models. In addition, we also performed stratified analyses by age groups (60–69 vs. 70 and over) and gender strata.

Finally, we performed two sensitivity analyses. First, in order to confirm the association of late-life depressive symptoms with MCI and to increase comparability with previous studies, we excluded participants below age of 65 (*n* = 4,423), leaving 9,808 subjects for the analysis. Second, to minimize reverse causality, we excluded participants who developed MCI during waves 2–4 (*n* = 205), leaving 14,026 participants for the analysis. Stata 14.0 was used for all analyses.

## Results

### Baseline characteristics of participants

Table 1 presents the prevalence of SDSs and baseline characteristics of the study population (*n* = 14,231). The median follow-up time of the study sample was 9.8 years, 55.95% were aged <70 years, 54.72% were women, and 22.87% had SDSs. In total population, the median *z*-score was 0.08 in immediate recall, 0.08 in delayed recall, 0.02 in verbal fluency, and 0.06 in global cognition. By contrast, the *z*-scores for all cognitive domains were negative in individuals with SDSs, and positive among those with non-SDSs.

**Table 1.** Baseline characteristics of the study population by depression status over the 10-year follow-up, *N* (%)/median (IQR).

Factor	Total population *N* = 14,231	Non-SDSs 10,976 (77.13)	SDSs 3,255 (22.87)
Follow-up time (years)	9.8 (6.2, 10.3)	9.9 (6.3, 10.3)	8.3 (6.1, 10.3)
Age (years)	68 (63, 74)	68 (63, 74)	70 (64, 76)
<70	7,962 (55.95)	6,346 (57.82)	1,616 (49.65)
≥70	6,269 (44.05)	4,630 (42.18)	1,639 (50.35)
Gender			
Male	6,444 (45.28)	5,517 (50.26)	927 (28.48)
Female	7,787 (54.72)	5,459 (49.74)	2,328 (71.52)
Marital status and living arrangement^1^			
Living with a partner	10,191 (72.09)	8,092 (74.27)	2,099 (64.78)
Living alone	3,945 (27.91)	2,804 (25.73)	1,141 (35.22)
Educational attainment			
Low	7,437 (52.26)	5,331 (48.57)	2,106 (64.70)
Middle	4,192 (29.46)	3,428 (31.23)	764 (23.47)
High	2,602 (18.28)	2,217 (20.20)	385 (11.83)
Smoking^1^			
Non/ex-smokers	12,219 (86.20)	9,375 (85.72)	2,844 (87.83)
Current-smokers	1,956 (13.80)	1,562 (14.28)	394 (12.17)
Alcohol assumption^1^			
None-drinkers	7,583 (53.51)	5,465 (49.98)	2,118 (65.45)
Usual-drinkers	3,277 (23.13)	2,757 (25.22)	520 (16.07)
Frequent-drinkers	3,310 (23.36)	2,712 (24.80)	598 (18.48)
Physical activity^1^			
Vigorous or moderate	12,747 (89.98)	10,217 (93.47)	2,530 (78.21)
Never	1,419 (10.02)	714 (6.53)	705 (21.79)
BMI^1^			
Normal	4,818 (34.67)	3,799 (35.36)	1,019 (32.31)
Underweight	113 (0.81)	79 (0.74)	34 (1.08)
Overweight	6,208 (44.67)	4,876 (45.39)	1,332 (42.23)
Obese	2,758 (19.85)	1,989 (18.51)	769 (24.38)
Number of chronic diseases			
0	2,766 (19.43)	2,505 (22.82)	261 (8.02)
1	4,148 (29.15)	3,476 (31.67)	672 (20.64)
2–3	5,329 (37.45)	3,923 (35.74)	1,406 (43.20)
≥4	1,988 (13.97)	1,072 (9.77)	916 (28.14)
History of affective or emotional disorders^1^			
No	13,114 (92.18)	10,520 (95.85)	2,594 (79.82)
Yes	1,112 (7.82)	456 (4.15)	656 (20.18)
Anti-anxiety or depression medication^1^			
No	13,470 (94.70)	10,625 (96.86)	2,845 (87.43)
Yes	754 (5.30)	345 (3.14)	409 (12.57)
Immediate recall^2^	0.08 (−0.53, 0.69)	0.15 (−0.43, 0.74)	−0.10 (−0.64, 0.54)
Delayed recall^2^	0.08 (−0.61, 0.75)	0.15 (−0.46, 0.82)	−0.18 (−0.76, 0.50)
Verbal fluency^2^	0.02 (−0.58, 0.66)	0.05 (−0.49, 0.68)	−0.20 (−0.73, 0.41)
Global cognition^2^	0.06 (−0.42, 0.58)	0.13 (−0.37, 0.62)	−0.16 (−0.61, 0.38)

Abbreviations: BMI, body mass index; IQR, interquartile range; *N*, numbers; SDSs, significant depressive symptoms.

1 Participants with missing values (n = 95 in marital status and living arrangement, 56 in smoking, 61 in alcohol assumption, 65 in physical activity, 334 in BMI, 5 in history of affective or emotional disorders, 7 in anti-anxiety or depression medication).

2 All cognitive scores are presented in standardized score.

In addition, compared with nonparticipants due to missing information, the study sample is significantly healthier as they were younger and had higher levels of education, less likely to be physically inactive and obese, less likely to have chronic diseases, history of affective or emotional disorders, and less likely to use anti-anxiety or depression medication, although more likely to drink alcohol and be overweight. Besides, they had lower EURO-D scores and better cognitive performance (Supplementary Table S1).

### Depressive symptoms and incident MCI from the Cox Proportional Hazards model

After an average of 8.2-year follow-up (SD: 2.4 years, range: 2.7–11.3 years), 1,352 individuals developed MCI (9.50%). Table 2 displays the HRs for incident MCI in people with SDSs compared those with non-SDSs. In total population, people with SDSs were at a significantly higher risk of incident MCI (HR = 1.26) after adjusting for potential confounders (age, gender, country, marital status and living arrangement, educational attainment, smoking, alcohol consumption, physical activity, BMI, the number of chronic diseases, history of affective or emotional disorders, and anti-anxiety or depression medication). The significantly increased risk is only present among individuals aged 70+ (HR = 1.35) and among women (HR = 1.28). These results were also confirmed when excluding participants below age of 65 (*n* = 4,423; Supplementary Table S2).

**Table 2.** Hazard ratios (95% confidence intervals) for incident MCI according to baseline depressive symptoms, and stratified by age (under 70 and 70 and over) and gender.

	Normal cognition *N* (%)	MCI *N* (%)	HR (95 CI%)
Total population			
Non-SDSs	10,066 (78.16)	910 (67.31)	1.00
SDSs	2,813 (21.84)	442 (32.69)	**1.26 (1.10–1.44)**
Age (years)			
<70			
Non-SDSs	5,933 (80.36)	413 (71.33)	1.00
SDSs	1,450 (19.64)	166 (28.67)	1.21 (0.98–1.49)
≥70			
Non-SDSs	4,133 (75.20)	497 (64.29)	1.00
SDSs	1,363 (24.80)	276 (35.71)	**1.35 (1.14–1.61)**
Gender			
Male			
Non-SDSs	5,060 (86.17)	457 (79.90)	1.00
SDSs	812 (13.83)	115 (20.10)	1.21 (0.96–1.52)
Female			
Non-SDSs	5,006 (71.44)	453 (58.08)	1.00
SDSs	2,001 (28.56)	327 (41.92)	**1.28 (1.08–1.51)**

Bold values indicate a significance level of *p* < 0.05.

HRs were obtained from Cox Proportional Hazards model adjusted for age, gender, country, marital status and living arrangement, educational attainment, smoking, alcohol consumption, physical activity, BMI, the number of chronic diseases, history of affective or emotional disorders and anti-anxiety or depression medication.

Abbreviations: CI, confidence intervals; BMI, body mass index; HR, hazard ratio; MCI, mild cognitive impairment; SDSs, significant depressive symptoms.

### Dose–response association between the severity of depressive symptoms and MCI incidence

**Figure 2.**
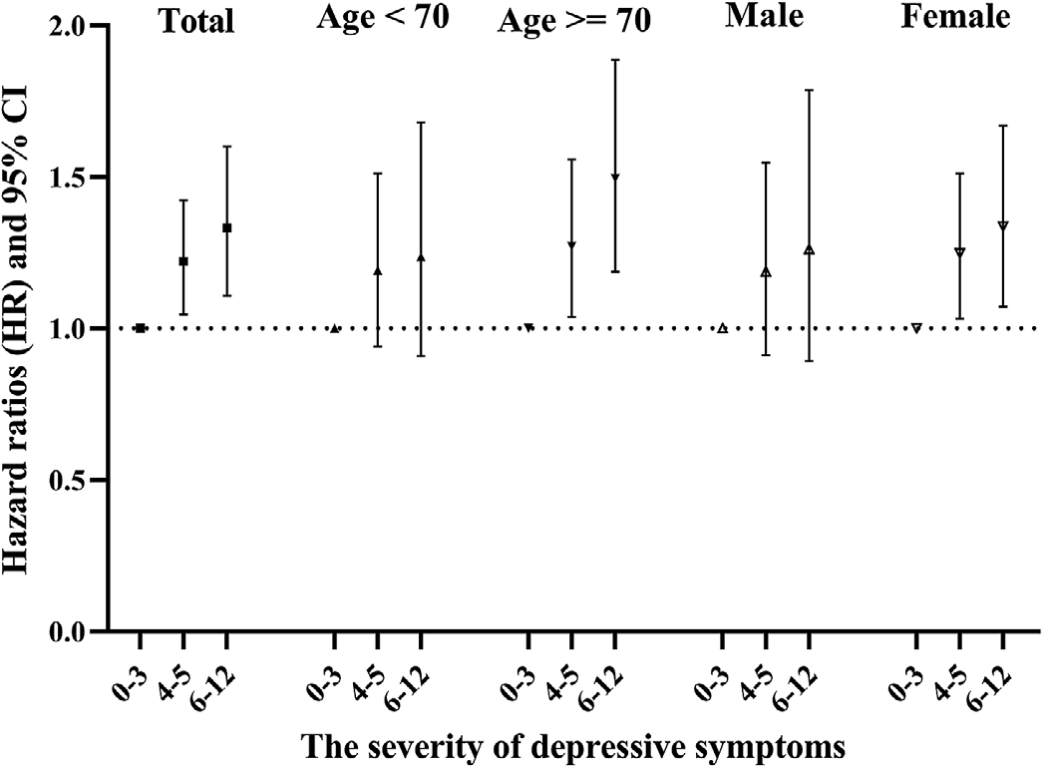
HRs were obtained from Cox Proportional Hazards model adjusted for age, gender, country, marital status and living arrangement, educational attainment, smoking, alcohol consumption, physical activity, body mass index (BMI), the number of chronic diseases, history of affective or emotional disorders and anti-anxiety or depression medication.

As shown in Figure 2, in total population, compared with no/minimal depressive symptoms (score 0–3), individuals with higher EURO-D scores had a significantly higher risk of incident MCI (*p*-trend <0.001) after adjustment for covariates, indicating a dose–response association. Stratified analyses by age and gender showed that the dose–response association was only observed among people aged ≥70 years and women. Among people aged ≥70 years, compared to those with no/minimal depressive symptoms, individuals with moderate depressive symptoms were at a significantly high risk of MCI (HR = 1.27) and this risk was even higher (HR = 1.50) among those with severe depressive symptoms. Among women, significant increased MCI risk were associated with moderate (HR = 1.25) and severe depressive symptoms (HR = 1.34) compared to no/minimal depressive symptoms. These results remained after excluding new MCI cases that were developed between waves 2 and 4 (*n* = 205; Supplementary Table S3).

## Discussion

This study provided evidence of an independent negative effect of SDSs on risk of incident MCI over a 10-year follow-up in total population, older participants (≥70 years) and women. When the severity of depressive symptoms was considered, there was a dose–response relationship: the more severe the depressive symptoms, the higher the incidence of MCI. Further stratified analysis revealed that the dose–response relationship was only retained among older participants (≥70 years) and women.

The prevalence of SDSs among those without MCI in the current study was 23%, which is consistent with a recent systematic review reporting a range of 2.8–35% in older adults [52]. However, the value was 31.3% among aged over 45 years Chinese, which was higher but within the range [53]. Another study reported a prevalence of depression was 11.8% among people aged over 65 years in Portugal [54]. Studies of the prevalence of SDSs differed greatly with regard to the characteristics of study population, rating scales, and diagnostic criteria [55]. In addition, the data in SHARE documented that there was a very strong heterogeneity among countries [56].

Our result that baseline SDSs were associated with increasing subsequent MCI incidence is consistent with most previous studies [18,57,58]. Conversely, a small number of studies have shown that depressive symptoms was not associated with cognitive impairment [59,60]. These inconsistent findings possibly due to the differences on the chosen cognitive tests and the definition of MCI. There are several possible interpretations for the relationship between depressive symptoms and cognitive impairment. First, SDSs may be a risk factor for cognitive impairment. Second, SDSs is a precursor of cognitive impairment. Third, SDSs and cognitive impairment simultaneously exist [61]. To date, most studies support the hypothesis that depressive symptom is both a risk factor and a precursor of cognitive impairment [62,63], our study confirmed the former.

Our finding demonstrated that there was a dose–response relationship between the severity of depressive symptoms and MCI incidence. This finding remained when excluding the new MCI cases developed during the first two waves’ follow-up and when excluding participants younger than 65 years. Similar findings were reported in a study showing that the risk of cognitive impairment increased for each 10-point increment on the Center for Epidemiologic Studies Depression Scale [64], but this relationship was not found in another cohort study [31]. Such inconsistence may be attributed to the differences in the assessment scales for depressive symptoms, follow-up time, study design, sample size, and sources of study population (population-based versus hospital-based).

Age-stratified analyses concerning the severity of depressive symptoms and MCI incidence are rare. Our results showed that a dose–response association was only observed among people aged 70+ years. These findings are in line with a study from Chilean, which reported that depressive symptoms were positively associated with incident MCI only among people older than 65 years [65]. In contrast, another previous study documented that this association was stronger and more stable among those <75 years [66]. These age specific associations need to be further studied because of variations in age cut-offs. This age-specific association observed in our study among those aged 70+ (or 65+) may be explained by the fact that retirees may face environmental changes, reduce the feelings of social participation, coupled with difficulty adapting to the life after retirement, are more susceptible to loneliness and emotional disorders, thus aggravating the effect of depressive symptoms on risk of MCI. In contrast, by the time of retirement transition (aged 60–65), people may aspire to retirement and have positive emotions to deal with their work and life, which may counteract the negative effect of depressive symptoms on risk of MCI.

Sex-stratified analyses of the link between the severity of depressive symptoms and MCI risk have yielded mixed results. Some studies found that the effect was specific to female [67], while others reported it was specific to male [65] or mild in men and severe in women [68]. Our findings that an independent negative association was only observed among women and in a dose–response manner are plausible because men and women differ in their neural structure and system. It has been shown that women had less intelligence-related gray matter and are more susceptible to the pathology of white matter than men in the course of dementia [69]. Besides, women were more anxious and emotional when facing with retirement changes [70]. Further, women have weak stress resistance, lower stress threshold, and are more prone to suffer from SDSs. By contrast, men are less sensitive to changes in mood and have a higher stress threshold, which may reduce their risk of MCI after experienced depressive symptoms.

The potential mechanisms between depressive symptoms and the risk of cognitive impairment involve endocrine system, inflammatory processes, and comorbidities. Depressive symptoms stimulate the production of glucocorticoid or deposition of beta-amyloid by activating the hypothalamic–pituitary–adrenal axis, which in turn cause damage to the hippocampus and lead to cognitive impairment [39]. Depressive symptoms may not only increase the secretion of pro-inflammatory cytokines [40,41], such as interleukin-6 and high sensitive c-reactive protein [71], relating to microglial activation, blood–brain barrier dysfunction, and subsequent neuronal damage [72], but also decrease the release of neurotrophic factors [73], maintaining neuronal health and regulating synaptic plasticity, which in combination could lead to cognitive impairment [74]. Moreover, some evidence suggest that this association could potentially be mediated by other comorbidities, particularly cardiovascular diseases, according to the “vascular-depression-dementia hypothesis” [75,76]. Although these hypotheses have been proposed, the exact biological mechanism of the link between depressive symptoms and cognitive impairment remains unclear, further research is needed to provide more evidence.

Strengths of the present study include: first, this is a prospective cohort study, with a 10-year follow-up, which may yield more reliable results. Second, the study population is a representative sample of older Europeans, which increased the generalizability of the findings. Third, we included those who participated in any of the follow-ups, which greatly increased the sample size and minimized selection bias. Fourth, Cox proportional hazard model was used to take into account the influence of follow-up time and the proportional hazard assumption was assessed, so that it would lead to better accurate and more reliable results than methods that do not consider the time influence or studies that used Cox regression models but did not examine the proportional hazard assumption. Fifth, we explored the influence of depressive symptoms on MCI, the stage of early cognitive decline, which has profound implications for the prevention of cognitive impairment. Finally, sensitivity analysis revealed similar results, which further enhanced the authenticity and reliability of the study.

Nonetheless, this study has several limitations. One notable drawback is lost to follow-up, people with severe cognitive impairments often drop out of the survey early, resulting in underestimating the association. In addition, depressive symptoms were ascertained by self-reported questionnaires rather than structured clinical diagnoses or objective measures, which may lead to informational bias. Another limitation is that we only considered the baseline depressive symptoms, but did not take into account the dynamic changes in depressive symptoms, which may influence the observed results. Although a number of confounders have been taken into account, other unmeasured confounders, such as genetic factors, inflammatory markers, and other unknown diseases may influence the results. Finally, compared with nonparticipants due to missing information, the study sample was healthier, which may underestimate the observed association between depressive symptoms and MCI incidence.

## Conclusions

Individuals with significant depressive symptoms were at significantly increased risk of subsequent MCI in a dose–response manner, especially among those aged 70+ years and women. These findings suggest that well-managed depressive symptoms treatment among people aged 70+ years and women may be effective to prevent MCI.

## Figures and Tables

**Figure:** click here to view supplementary material

## Data Availability

This study used data from the Survey of Health, Ageing and Retirement in Europe, which is freely available to academic researchers (http://www.share-project.org).

## Supplementary Materials

To view supplementary material for this article, please visit http://dx.doi.org/10.1192/j.eurpsy.2021.2230.
