# Supplementary material for: Depressive symptoms and cognitive impairment: A 10-year follow-up study from the Survey of Health, Ageing and Retirement in Europe
Source: Eur Psychiatry. 2021 Aug 27;64(1):e55. doi: 10.1192/j.eurpsy.2021.2230 (PMC8446071; doi:10.1192/j.eurpsy.2021.2230)
Supplement: Supplementary file 1 [file S0924933821022306sup001.docx]

**Supplementary Material**

Table S1. Characteristics of the study sample and drop-outs due to missing information, N (%)/Mean ± SD

| Factor | Study sample N = 14231 (80.72) | Drop-outs N = 3399 (19.28) | *P*-value |
| --- | --- | --- | --- |
| Age (y) | 69.38 ± 7.04 | 72.09 ± 8.63 | <0.001 |
| Female | 7787 (54.72) | 1825 (54.35) | 0.70 |
| Living alone | 3945 (27.91) | 1219 (38.08) | <0.001 |
| Educational attainment |  |  | <0.001 |
| Low | 7437 (52.26) | 752 (65.68) |  |
| Middle | 4192 (29.46) | 249 (21.75) |  |
| High | 2602 (18.28) | 144 (12.58) |  |
| Current-smokers | 1956 (13.80) | 472 (14.36) | 0.40 |
| Alcohol consumption |  |  | <0.001 |
| None-drinkers | 7583 (53.51) | 2092 (63.72) |  |
| Usual-drinkers | 3277 (23.13) | 592 (18.03) |  |
| Frequent-drinkers | 3310 (23.36) | 599 (18.25) |  |
| Physically inactive | 1419 (10.02) | 739 (22.53) | <0.001 |
| BMI |  |  | <0.001 |
| Normal | 4818 (34.67) | 1045 (34.23) |  |
| Underweight | 113 (0.81) | 49 (1.60) |  |
| Overweight | 6208 (44.67) | 1278 (41.86) |  |
| Obese | 2758 (19.85) | 681 (22.31) |  |
| Number of chronic diseases |  |  | <0.001 |
| 0 | 2766 (19.44) | 511 (15.52) |  |
| 1 | 4148 (29.15) | 910 (27.64) |  |
| 2-3 | 5329 (37.45) | 1275 (38.73) |  |
| ≥ 4 | 1988 (13.97) | 596 (18.10) |  |
| History of affective or emotional disorders | 1112 (7.82) | 323 (10.10) | <0.001 |
| Anti-anxiety or depression medication | 754 (5.30) | 207 (6.24) | 0.032 |
| EURO-D scores | 2.19 ± 2.15 | 2.57± 2.53 | <0.001 |
| SDSs | 3255 (22.87) | 806 (29.07) | <0.001 |
| Immediate recall^#^ | 0.11 ± 0.91 | 0.05 ± 0.95 | 0.001 |
| Delayed recall^#^ | 0.10 ± 0.97 | 0.02 ± 0.98 | <0.001 |
| Verbal fluency^#^ | 0.09 ± 0.96 | 0.02 ± 0.96 | 0.001 |
| Global cognition^#^ | 0.10 ± 0.74 | 0.02 ± 0.79 | <0.001 |

N: numbers. SD: standard deviation. SDSs: significant depressive symptoms.

^#^ All cognitive scores are presented in standardized score

Table S2. Hazard ratios (HR) and 95% confidence interval (CI) of incident MCI in relation to baseline depressive symptoms, (n = 9808, after excluding 4423 participants below age of 65)

|  | Normal cognition N (%) | MCI N (%) | HR (95% CI) |
| --- | --- | --- | --- |
| Total population |  |  |  |
| Non-SDSs | 6737 (76.77) | 682 (66.09) | 1.00 |
| SDSs | 2039 (23.23) | 350 (33.91) | **1.23 (1.05-1.43)** |
| EURO-D score |  |  |  |
| 0~3 | 6737 (76.77) | 682 (66.09) | 1.00 |
| 4~5 | 1258 (14.33) | 185 (17.93) | 1.17 (0.98-1.40) |
| 6~12 | 781 (8.90) | 165 (15.99) | **1.32 (1.08-1.62)** |
| Stratified by age |  |  |  |
| <70 |  |  |  |
| Non-SDSs | 2604 (79.39) | 185 (71.43) | 1.00 |
| SDSs | 676 (20.61) | 74 (28.57) | 1.02 (0.74-1.40) |
| EURO-D score |  |  |  |
| 0~3 | 2604 (79.39) | 185 (71.43) | 1.00 |
| 4~5 | 434 (13.23) | 41 (15.83) | 1.01 (0.70-1.45) |
| 6~12 | 242 (7.38) | 33 (12.74) | 1.08 (0.68-1.70) |
| >=70 |  |  |  |
| Non-SDSs | 4133 (75.20) | 497 (64.29) | 1.00 |
| SDSs | 1363 (24.80) | 276 (35.71) | **1.35 (1.14-1.61)** |
| EURO-D score |  |  |  |
| 0~3 | 4133 (75.20) | 497 (64.29) | 1.00 |
| 4~5 | 824 (14.99) | 144 (18.63) | 1.22 (1.00-1.50) |
| 6~12 | 539 (9.81) | 132 (17.08) | **1.42 (1.13-1.79)** |

MCI: mild cognitive impairment. SDSs: significant depressive symptoms. EURO-D: Europe-depression scale.

Bold values indicate a significance level of *P* < 0.05.

HRs were obtained from Cox Proportional Hazards model adjusted for age, gender, country, marital status and living arrangement, educational attainment, smoking, alcohol consumption, physical activity, BMI, the number of chronic diseases, history of affective or emotional disorders and anti-anxiety or depression medication.

Table S3. Hazard ratios (HR) and 95% confidence interval (CI) of MCI incidence in relation to baseline depressive symptoms (n=14026, after excluding 205 cases who developed MCI between waves 2 to 4)

|  | Normal cognition N (%) | MCI N (%) | HR (95% CI) |
| --- | --- | --- | --- |
| Total population |  |  |  |
| Non-SDSs | 10066 (78.16) | 781 (68.09) | 1.00 |
| SDSs | 2813 (21.84) | 366 (31.91) | **1.18 (1.02-1.37)** |
| EURO-D score |  |  |  |
| 0~3 | 10066 (78.16) | 781 (68.09) | 1.00 |
| 4~5 | 1756 (13.70) | 201 (17.52) | 1.16 (0.98-1.37) |
| 6~12 | 1048 (8.14) | 165 (14.39) | **1.23 (1.01-1.50)** |

MCI: mild cognitive impairment. SDSs: significant depressive symptoms. EURO-D: Europe-depression scale.

Bold values indicate a significance level of *P* < 0.05.

HRs were obtained from Cox Proportional Hazards model adjusted for age, gender, country, marital status and living arrangement, educational attainment, smoking, alcohol consumption, physical activity, BMI, the number of chronic diseases, history of affective or emotional disorders and anti-anxiety or depression medication.
